# Construction of SNP fingerprints and genetic diversity analysis of radish (*Raphanus sativus* L.)

**DOI:** 10.3389/fpls.2024.1329890

**Published:** 2024-02-02

**Authors:** Xiaolin Xing, Tianhua Hu, Yikui Wang, Yan Li, Wuhong Wang, Haijiao Hu, Qingzhen Wei, Yaqin Yan, Defang Gan, Chonglai Bao, Jinglei Wang

**Affiliations:** ^1^ School of Horticulture, Anhui Agricultural University, Hefei, China; ^2^ Institute of Vegetables, Zhejiang Academy of Agricultural Sciences, Hangzhou, China; ^3^ Institute of Vegetables, Guangxi Academy of Agricultural Sciences, Nanning, China

**Keywords:** radish, SNP, KASP, fingerprint, genetic diversity

## Abstract

Radish (*Raphanus sativus* L.) is a vegetable crop with economic value and ecological significance in the genus Radish, family *Brassicaceae*. In recent years, developed countries have attached great importance to the collection and conservation of radish germplasm resources and their research and utilization, but the lack of population genetic information and molecular markers has hindered the development of the genetic breeding of radish. In this study, we integrated the radish genomic data published in databases for the development of single-nucleotide polymorphism (SNP) markers, and obtained a dataset of 308 high-quality SNPs under strict selection criteria. With the support of Kompetitive Allele-Specific PCR (KASP) technology, we screened a set of 32 candidate core SNP marker sets to analyse the genetic diversity of the collected 356 radish varieties. The results showed that the mean values of polymorphism information content (PIC), minor allele frequency (MAF), gene diversity and heterozygosity of the 32 candidate core SNP markers were 0.32, 0.30, 0.40 and 0.25, respectively. Population structural analysis, principal component analysis and genetic evolutionary tree analysis indicated that the 356 radish materials were best classified into two taxa, and that the two taxa of the material were closely genetically exchanged. Finally, on the basis of 32 candidate core SNP markers we calculated 15 core markers using a computer algorithm to construct a fingerprint map of 356 radish varieties. Furthermore, we constructed a core germplasm population consisting of 71 radish materials using 32 candidate core markers. In this study, we developed SNP markers for radish cultivar identification and genetic diversity analysis, and constructed DNA fingerprints, providing a basis for the identification of radish germplasm resources and molecular marker-assisted breeding as well as genetic research.

## Introduction

Radish (*Raphanus sativus* L., 2n = 2× = 18) is an annual or biennial herb in the genus *Raphanus* of the *Brassicaceae* family capable of forming large, fleshy roots, originating from the wild radish (*Raphanus raphanistrum* L.) on the warm shores of Eurasia, it is an important vegetable crop in much of the world (Kang et al., 2016). Radishes are rich in vitamins and nutrients and are eaten in a variety of ways, making their unique flavour extremely popular in the human diet (Noman et al., 2021). In China, the cultivated area of radish is as high as 1.2 million square hectares, with an annual output of more than 40 million tons. Moreover, China is rich in radish germplasm resources, and has collected and preserved 2 073 domestic and foreign radish germplasm resources, with 95% of the preserved resources being domestic local varieties (Wang et al., 2018). Therefore, China occupies an important position in the collection and preservation of radish germplasm resources and research. The radish is one of the oldest cultivated crops, and during its spread different variants have evolved worldwide, such as *Raphanus sativus* L. var. *radiculuse* Pers, *Raphanus sativus* L. var. *oleifera*, *Raphanus sativus* L. var. *caudatus*, *Raphanus sativus* L. var. *niger*, and *Raphanus sativus* L. var. *longipinnatus* Bailey (Li et al., 2021). Due to the frequent introduction of seeds from different regions, the difficulty of preserving local variety resources, and the increasing number of new varieties selected and bred, the phenomenon of different species have been assigned the same name or different names have been assigned a single species inevitably occurs, which greatly increases the difficulty of radish variety identification (Jamali et al., 2019). Therefore, it is of great significance to establish an economical, efficient and accurate radish variety identification method to improve the research and conservation of radish genetic resources, variety identification and sustainable development of the radish industry.

Radish germplasm resources are an important material basis for the selection and breeding of new radish varieties, genetic background research and agricultural production (Selvakumar, 2022). For breeding workers, it is very important to clarify the genetic relationship between germplasm resources and breeding materials. The traditional identification method is mainly morphological identification, but it is susceptible to the external environment and human subjective influence (Hong et al., 2023). In recent years, with the development of molecular biology and genomics, DNA molecular markers have been widely used in genetic diversity, kinship and fingerprinting studies of various crops (Nadeem et al., 2018). Among them, simple sequence repeat (SSR) and single-nucleotide polymorphism (SNP) molecular markers have been prioritized and recommended by the International Union for the Protection of New Plant Variety Rights (UPOV) (Button, 2007). SNP molecular markers, as the most mainstream third-generation molecular markers, are widely used in crop variety identification and map construction because of their wide quantitative distribution, high polymorphism and good stability (Wang et al., 2021). Kompetitive Allele Specific PCR (KASP) is a new type of high-throughput SNP typing technology, what has high accuracy, high throughput, and low cost, and is currently the most ideal genotyping technology (Semagn et al., 2014). It has been widely applied to crops such as rice (Chen et al., 2014; Yang et al., 2019), wheat (Grewal et al., 2020a; Dreisigacker et al., 2019; Grewal et al., 2020b), maize (Chen et al., 2021; Tian et al., 2021), cucumber (Zhang et al., 2020) and grape (Wang F. et al., 2022).

DNA fingerprinting refers to the use of molecular markers to construct a DNA profile that can directly reflect the differences between biological individuals at the DNA molecular level (Sucher et al., 2012). Due to the large number of molecular markers and their high polymorphism, they are widely distributed in the genome; they can be detected at different stages of growth and development; and they can identify the pure and heterozygous genotypes of crop varieties or lines, providing great convenience for genetic breeding (Nybom et al., 2014). Therefore, DNA fingerprinting technology is widely used in the identification and analysis of kinship between crop varieties (Yang et al., 2019; Wang L. et al., 2022), seed purity testing (Satturu et al., 2018) protection of varietal rights and interests (Iqbal et al., 2021; Jamil et al., 2021) and other research. Over the past two decades, breeders have analysed the genetic diversity of radish separately using random amplified polymorphic DNA (RAPD), SSR, and structure variation (SV), and constructed core germplasm resources to lay the foundation for the genetic breeding of radish (Pradhan et al., 2004; Bae et al., 2015; Li et al., 2023). In recent years, fingerprinting based on SNP construction has been accomplished in most crops (Tian et al., 2015; Wang et al., 2021; Ongom et al., 2021), especially cruciferous vegetables. In Chinese cabbage, Li et al. (2019) analysed the genetic identification of 60 core SNPs screened with 178 commercial species and confirmed the practicality of the SNP markers. In broccoli, Shen et al. (2021) sequenced the whole genomes of 23 representative broccoli varieties, and developed and identified 28 KASP markers for broccoli varietal fingerprinting. Further studies on the genetic diversity, genetic relationships and population structure of 372 broccoli materials showed that the broccoli population had a limited genetic background. In cauliflower, Yang et al. (2022) identified 41 highly polymorphic SNPs and constructed 329 cauliflower DNA fingerprints. However, thus far, no SNP-based fingerprint database for radish has been reported.

Radish is a typical heterogamous pollinated plant with strong self-incompatibility (Huang et al., 2023), its planting area is very extensive, and seed retention is uneven in different places, therefore it is difficult to maintain the purity of the seeds, and even the varieties are degraded, leading to ambiguous genealogical relationships (Pearse et al., 2014). Thus, there is an urgent need to establish a fast, accurate and simple method to improve the identification of radish germplasm resources and their breeding efficiency. KASP technology is fast and accurate for SNP detection and analysis (Semagn et al., 2014); this technology can therefore be used to genotype the core SNPs of radish germplasm resources, construct the main varieties of DNA fingerprints, elucidate the genetic diversity of radish, which is conducive to promoting molecular genetic breeding, and protect the breeding of radish.

In this study, we used radish genome sequencing data to develop a core SNP database for radish cultivar identification. Using 46 representative radish cultivars for screening, we obtained a set of 32 candidate markers with polymorphisms that were evenly distributed throughout the radish genome. Subsequently, we genotyped 356 radish varieties based on KASP technology and obtained genotype data. Based on these genotyping results, the core markers for radish germplasm fingerprinting were screened and DNA fingerprinting was performed. We also analysed the genetic diversity and variety identification of these 356 varieties based on the KASP genotyping results. Our results not only provide new molecular markers for radish variety identification, but also lay the foundation for the conservation and innovation of radish germplasm resources in the future.

## Materials and methods

### Plant materials and DNA extraction

In this study, we collected 356 radish materials for DNA fingerprinting (**Supplementary Table 1**), and screened for candidate SNPs using 46 representative radish varieties (**Supplementary Figure 1**, **2**; **Supplementary Table 2**), which included local cultivars, F1 hybrids, and commercial varieties purchased from the market. Some materials were also provided by the Vegetable Institute of Zhejiang Academy of Agricultural Sciences and commercial breeding companies.

Two fresh young leaves with diameters of 1 cm were collected, and steel beads approximately 4 mm in diameter were added. After sufficient freezing in liquid nitrogen, the samples were ground into powder form using a tissue grinder, and genomic DNA was extracted using the CTAB method (Aboul-Maaty and Oraby, 2019).The DNA concentration of the samples to be tested was measured, the quality of the samples was checked using the BioDrop uLite Nucleic Acid Micromanometer, and the concentration was adjusted to 10–20 ng/μL with sterile water.

### SNP discovery and selection

To obtain universal SNPs for radish cultivar identification, we used eight whole-genome resequencing materials provided by the Vegetable Institute of Zhejiang Academy of Agricultural Sciences (stored in CNGBdb under the accession number CNP0004871 and in NCBI under the accession number PRJNA836770) (Wei et al., 2022) and 520 radish simplified genome sequencing data published in the DDBJ database (accession numbers DRA008624, DRA008625, DRA008636, and DRA008637) (Kobayashi et al., 2020) for molecular marker development. The genomic data were first subjected to the removal of low-quality sequences and adapters using FASTP (Chen et al., 2018) software, and then the QC-controlled data were aligned to the radish reference genome Xinlimei (Zhang et al., 2021), which has a genome assembly size of 460 Mb and a Contig N50 length of 18.72 Mb using BWA (Li and Durbin, 2009) software. The resulting sequence alignment/mapping (SAM) format file was converted to a binary sequence alignment/map format file, and SNP calls were made using the mpileup and view options of samtools (Li et al., 2009). Duplicate reads were then removed using the MarkDuplicates function of GATK (McKenna et al., 2010); the original variants (SNPs and InDels) were generated using the HaplotypeCaller option; SNPs were separated using the SelectVariants option; and the original SNPs were filtered using the VariantFiltration option. The VariantFiltration option was used to filter the original SNPs and a database of DNA variants containing the radish SNPs was generated. In addition, we used Python scripts to filter 50 bp upstream and downstream of the obtained SNPs without other variant sites, and then filtered again according to standard parameter settings using vcftools (Petr et al., 2011) software. According to the filtering criteria, we adapted some parameter settings: (1) Coarse filtering was carried out using the GATK software (parameters: QD < 2.0, MQ < 40.0, MQRankSum < - 12.5, ReadPosRankSum < - 8.0); (2) Use a Python script to filter the SNP loci with no other variant loci at 50 bp before and after; and (3) Use VCFtools software to filter based on screening parameters (parameters: - max-alleles 2 - min-alleles 2 - max-missing 0.5 - mac 3 - minQ 30 - maf 0.05 - mean-DP 1.2). The high-quality SNPs ultimately obtained were used for candidate SNP screening, KASP marker design, fingerprint construction and population genetic analysis.

### KASP genotyping

Extracted sequences around candidate SNPs 100 bp upstream and downstream were used for KASP marker design. For each KASP target site, two allele-specific primers and one universal primer were designed. The primer design parameters were set as follows: (1) GC content <60%; (2) melting temperature (Tm) between 55 and 62°C; and (3) PCR product size not larger than 120 bp. The primers were synthesized by Zhejiang Yihe Genetics Co. Ltd. with FAM- or HEX-tails (FAM tail: 5′-GAAGGTGACCAAGTTCATGCT-3′; HEX tail: 5′-GAAGGTCGGGAGTCAACGGATT-3′). The KASP assay was performed in a 1.6 μL PCR system/condition that consisted of 0.8 μL of KASP Master mix (LGC, Biosearch Technologies), approximately equal to 0.05 μL of primer, and 0.8 μL of DNA at a concentration of 10–20 ng/μL. The PCR program was as follows: 15 min at 94 °C, 10 touchdown cycles of 94 °C for 20 s and 61–55 °C for 60 s (decreasing by 0.6 °C per cycle), and 26 cycles of 94 °C for 20 s and 55 °C for 60 s. After the PCR, fluorescence data were read and analysed using an IntelliQube machine (LGC, Biosearch Technologies).

### Data analysis

Genotyping data were organized using Excel 2016 software. Powernarker V3.25 (Liu and Muse, 2005) software was used to calculate genetic diversity parameters such as polymorphism information content (PIC), minimum allele frequency (MAF), GeneDiversity (GD), and heterozygosity (He). The two-by-two genetic distance matrix of genotyping data was calculated, based on which thr Nei1972 standard genetic distance-based neighbour-joining (NJ) tree was constructed and the resulting neighbour-joining tree was visualized using MEGA-X (Kumar et al., 2018) software. Tassel 5.1 (Bradbury et al., 2007) was used to carry out the principal component analysis (PCA). Bayesian clustering was performed using STRUCTURE 2.3.4 (Falush et al., 2003) and the best grouping of classes was determined. The parameters were set as follows: the burn-in period was 50 000, the number of MCMC repeats was set to 100 000, the optimal number of groups (k) was determined using the ΔK method (Δk: 1-20), and the number of iterations was 5. After running the software, the result file with suffix “_f” was compressed and uploaded to the online website “STRUCTURE HARVESTER” (http://taylor0.biology.ucla.edu/struct_harvest/) to determine the optimal K value according to the method of EVANNO et al. (Evanno et al., 2005).

### DNA fingerprinting

For the obtained SNP genotyping results, the optimal combination of markers was calculated for fingerprinting using SNPT software (http://www.shigatox.net/stec/cgi-bin/snpt). The genotypes of the optimal combination of markers were heatmapped for fingerprinting using RStudio, where each row of the heatmap represents one SNP locus and each column represents one sample. Among them, the pure genotypes are AA=yellow, CC=green, GG=blue, TT=purple; heterozygous genotypes are grey; and the no call genotypes are NA=white.

### Core collection development

In order to determine the representativeness of the genotypes of the core germplasm population of Chinese radish, Corehunter software (Thachuk et al., 2009) was used for evaluation and analysis, and the Rogers genetic distance (MR), Cavalli-Edwards genetic distance (CE), Shannon index (SH), expected heterozygosity (HE), polymorphic information PIC and allele coverage (CV) were calculated, and finally the optimal size of the core germplasm population of Chinese radish was determined.

## Results

### Development and identification of SNPs

**Figure 1:**
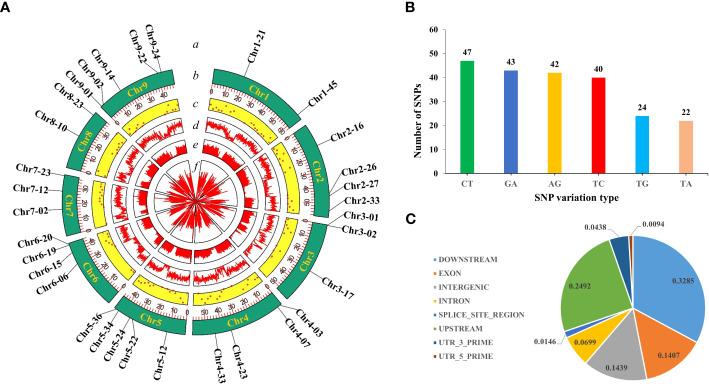
SNP screening and statistics. **(A)** Development of high-quality SNPs. From the outside to the inside: a, 32 highly polymorphic SNPs; b, number of chromosomes; c, Distribution of 308 high-quality SNPs; d, VCFtools filtering; e, Python script filtering; f, GATK filtering. **(B)** Genotype statistics for the six most commonly identified variants. **(C)** The positions of the SNPs in the gene structures.

In this study, we used 520 simplified radish genomic datasets (51.6 GB) and 8 resequencing datasets (31.8 GB) and mined a total of 10,540,000 SNPs. According to the filtering criteria, 308 high-quality SNPs were ultimately obtained, which were uniformly distributed on each chromosome of radish (**Figure 1A**).

The single-base variant type statistics of the obtained high-quality SNPs revealed that there were 12 variant types, indicating that the radish genome is rich in variant types. In particular, C/T (15%), G/A (14%), A/G (14%) and T/C (13%) were the top four genotypes with the highest proportions of all variant types, and the ratio of conversion to subversion was approximately 1.17 (**Figure 1B**). These studies indicated that the 308 high-quality SNPs were ideal for constructing radish fingerprints and performing genetic diversity analysis. In addition further analysis of the distribution of high quality SNPs across the genome revealed that 14.39% were located in intergenic regions, 14.07% were located in exons, 6.98% were located in introns, 24.92% were located in the 1-kb region upstream of the transcriptional start site, 32.84% were located in the 1-kb region downstream of the transcription termination site, 0.93 and 4.38% were located in the 5′ and 3′ UTRs, and 1.46% were located in the splice junctions (**Figure 1C**).

### KASP marker transformation and screening

**Figure 2:**
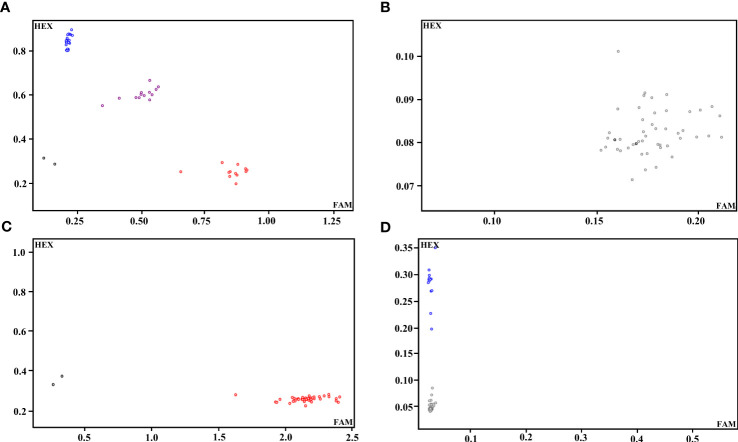
Representative KASP-labelled fluorescence assay results. **(A)** A KASP marker with better typing. **(B)** A KASP marker that was untypable. **(C)** A KASP marker with monomorphism. **(D)** A KASP marker with a high no call rate.

We selected 100 SNPs from 308 high-quality SNPs with 8-12 SNP loci evenly distributed on each chromosome of radish, and extracted 100 bp upstream and downstream sequences for KASP primer design. Thirteen SNPs could not be transformed into KASP markers due to the lack of sequence specificity or abnormal GC content. The remaining 87 SNPs (87% transformation rate) were successfully transformed into KASP markers, and 46 representative radish varieties with different traits (cotyledon, attitude of leaf fascicle, leaf shape, leaf color, leaf, incision, et al.) were utilized to validate the 87 KASP markers. According to the typing results, 19 markers could not be typed successfully, and 14 markers with monomorphism and 22 markers with >10% no call rate were excluded (**Figures 2A-D**); ultimately, 32 candidate core SNP markers were screened and used for genetic diversity analysis of radish germplasm (**Figure 1A**; **Supplementary Table 3**).

**Figure 3:**
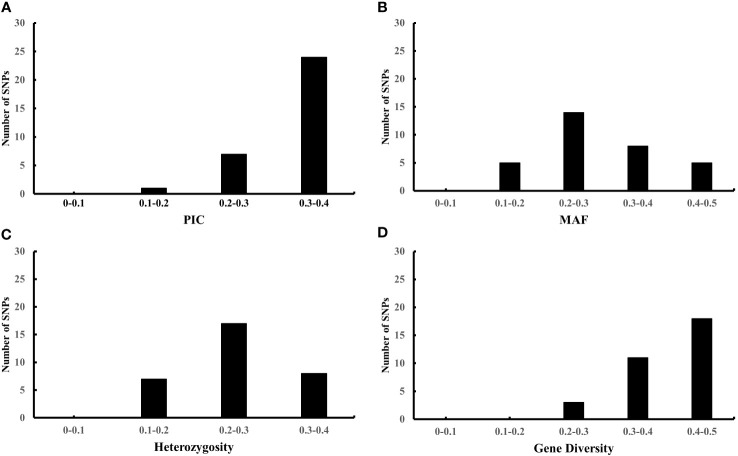
Population genetics analysis of 32 candidate core markers in 356 radish materials including PIC **(A)**, MAF **(B)**, heterozygosity **(C)**, and gene diversity **(D)**.

To further evaluate the effectiveness of the 32 candidate core SNPs in radish variety identification, we performed KASP analysis using 356 radish materials. The results showed that all 32 KASP markers had good typing results. Among them, the PIC values ranged from 0.195 to 0.375, with a mean value of 0.32, and 90% of the markers were highly polymorphic (PIC > 0.25) (**Figure 3A**). The MAF values ranged from 0.125 to 0.482, with means value of 0.303 (**Figure 3B**), and the heterozygosity and gene diversity values ranged between 0.1-0.4 and 0.2-0.5 with a mean of 0.253 and 0.406, respectively, indicating high genetic polymorphism (**Figures 3C, D**). Notably, all 32 candidate core SNP no call rates were below 10%, with 28 KASP markers having no call rates below 5% in 356 radish materials (**Table 1**). All these data indicated that the 32 candidate core SNPs were highly reliable as markers for genetic diversity analysis of radish germplasm resources.

**Table 1:** Genetic diversity information of 32 KASP markers in 356 radish materials.

Marker	Chromosome	Position	Variation type	PIC	MAF	GeneDiversity	Heterozygosity	Missing rate
Chr1-21	1	18753042	T/C	0.321	0.279	0.402	0.255	0.053
Chr1-45	1	58808047	T/A	0.327	0.290	0.412	0.225	0.065
Chr2-16	2	14156674	C/T	0.290	0.229	0.353	0.240	0.017
Chr2-26	2	44476246	G/A	0.292	0.231	0.356	0.234	0.017
Chr2-27	2	45748274	A/G	0.360	0.379	0.471	0.294	0.045
Chr2-33	2	54832993	G/T	0.368	0.414	0.485	0.260	0.081
Chr3-01	3	301586	A/G	0.354	0.358	0.459	0.365	0.053
Chr3-02	3	362352	G/A	0.324	0.283	0.406	0.123	0.087
Chr3-17	3	29949211	G/A	0.266	0.197	0.317	0.315	0.003
Chr4-03	4	1251819	T/A	0.374	0.471	0.498	0.257	0.039
Chr4-07	4	2932692	C/G	0.316	0.268	0.393	0.185	0.011
Chr4-23	4	34449708	C/T	0.304	0.249	0.374	0.280	0.017
Chr4-33	4	44369429	T/G	0.367	0.413	0.485	0.368	0.045
Chr5-12	5	11242018	A/G	0.359	0.376	0.469	0.334	0.034
Chr5-22	5	33498683	T/C	0.266	0.197	0.316	0.188	0.045
Chr5-24	5	35234907	T/C	0.315	0.268	0.392	0.277	0.067
Chr5-34	5	43324346	C/T	0.364	0.394	0.478	0.288	0.045
Chr5-36	5	44922478	G/A	0.195	0.125	0.219	0.148	0.011
Chr6-06	6	30694641	G/A	0.318	0.272	0.396	0.216	0.025
Chr6-15	6	38117650	A/G	0.355	0.362	0.462	0.313	0.003
Chr6-19	6	42865438	A/T	0.375	0.482	0.499	0.269	0.039
Chr6-20	6	43227414	T/A	0.350	0.346	0.453	0.297	0.006
Chr7-02	7	16504349	C/T	0.232	0.159	0.268	0.149	0.003
Chr7-12	7	27487519	G/C	0.316	0.269	0.393	0.305	0.014
Chr7-23	7	36882600	G/A	0.306	0.252	0.377	0.147	0.025
Chr8-10	8	20479094	G/A	0.360	0.379	0.471	0.249	0.028
Chr8-23	8	35858487	G/T	0.370	0.432	0.491	0.305	0.034
Chr9-01	9	652195	G/A	0.276	0.209	0.331	0.332	0.020
Chr9-02	9	1929299	A/G	0.321	0.278	0.401	0.211	0.039
Chr9-14	9	15485852	G/T	0.246	0.174	0.287	0.151	0.031
Chr9-22	9	40713031	C/T	0.361	0.383	0.472	0.273	0.042
Chr9-24	9	42576246	C/T	0.327	0.289	0.411	0.237	0.028

### Analysis of genetic diversity

**Figure 4:**
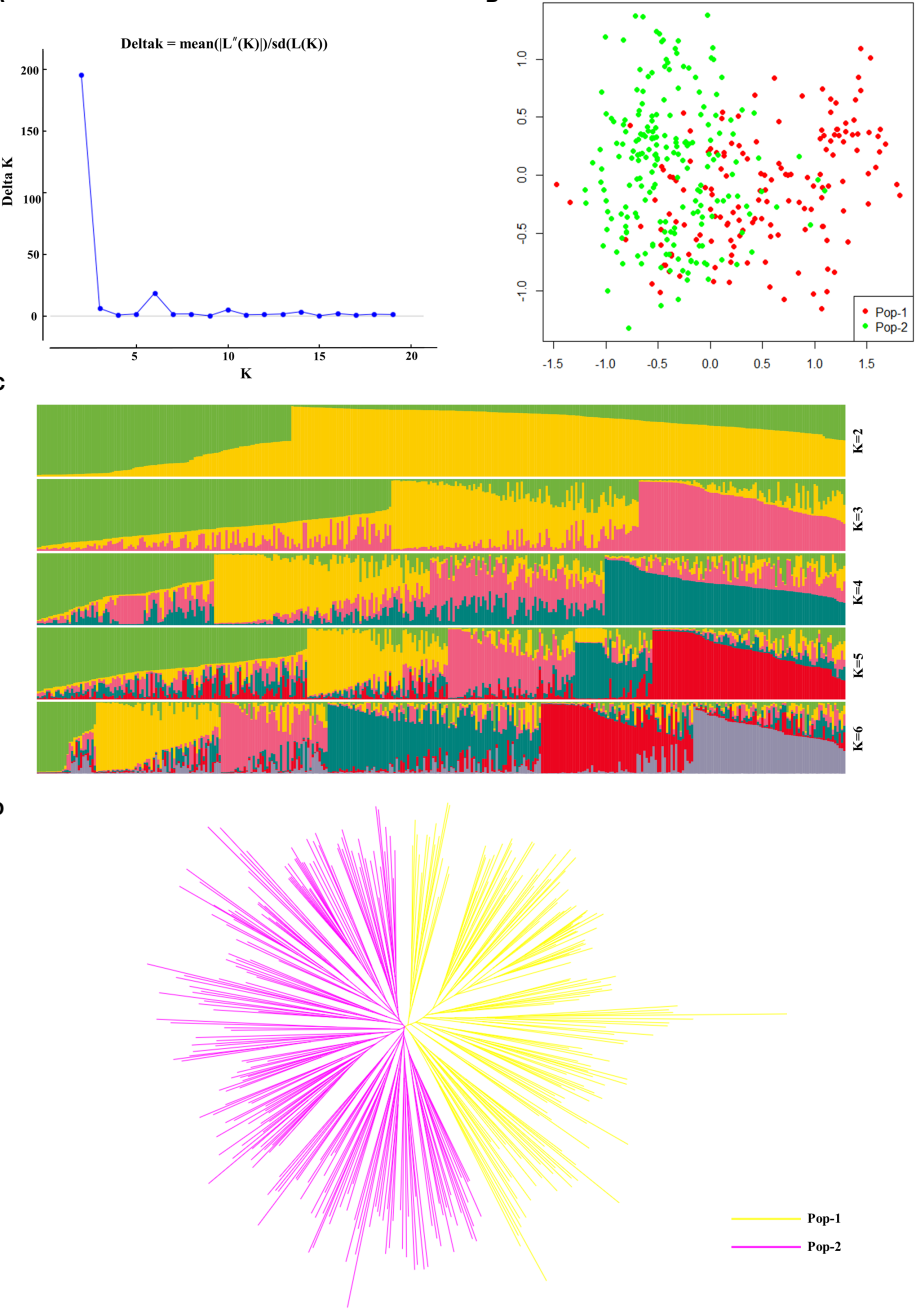
Genetic diversity analysis based on 32 candidate core SNP markers among 356 radish materials. **(A)** The ΔK values corresponding to different K measurements. **(B)** Principal component analysis of 356 radish materials. **(C)** Population structure of the 356 radish germplasm resources at different values of K. **(D)** Kinship tree of 356 radish materials based on principal component analysis.

In this study, we analysed the population structure of 356 radish materials based on the KASP typing results, and the optimal K value was analysed using the online website Structure Harvester (http://taylor0.biology.ucla.edu/struct_harvest/). The number of clusters is usually determined based on the cross-validation error rate, and the number of clusters with the lowest cross-validation error rate is the optimal number of clusters. The cross-validation error rate is the lowest when K=2, ΔK reached the maximum value, and it could be inferred that the 356 materials could be divided into two populations, Pop-1 and Pop-2, (**Figures 4A, C**). On the basis of the genotyping data, we further performed PCA, which showed that the 356 radish materials were clustered into two groups, Pop-1 (42.7%) and Pop-2 (57.3%), based on the first (PC1) and second (PC2) principal components. In the PCA two-dimensional schematic diagrams (**Figure 4B**), the two populations were revealed to be densely interacting with each other and both populations contained materials from different geographical locations, indicating that the 356 radish materials were closely genetically related to each other and that there was no significant correlation with the geographic origin of the materials. Further genetic analysis of the 356 radish materials showed that the 356 radish materials were divided into two subgroups, of which Pop-1 (yellow) contained 152 radish materials and Pop-2 (pink) contained 204 radish materials (**Figure 4D**). However, cherry radish and large-root radish were distributed in different branches, indicating that the genetic background of Chinese radish was complex, and different radish species were related to each other and independent of each other.

Furthermore, we used Power Marker software to calculate the population genetic distance (p-distance) (**Supplementary Table 4**) (Wang et al., 2021) for KASP genetic analysis data, calculated the genetic distance similarity coefficient Nie1972 (**Supplementary Table 5**) based on the generated genetic distance matrix, and then utilized the neighbour-joining method to construct a phylogenetic tree (**Supplementary Figure 3**). The results of the phylogenetic tree analysis were consistent with those of the population structure analysis and PCA, which classified the 356 radish populations into two categories, including Pop-1 (157, green) and Pop-2 (199, red). Interestingly, we found that seven of these materials clustered together in the phylogenetic tree analysis with genetic similarity coefficients of 0; these radish materials were Rs184, Rs187, Rs304, Rs315, Rs318, Rs320, and Rs321. We searched for their KASP genotyping results and found that the genotypes of the seven materials were almost but not exactly identical, their genotypic differences from each other are deletions of one genotype, as is Rs113 and Rs323.

Therefore, we speculate that these materials may be elite varieties of progeny selected from the breeding of the same varietal materials as their parents.More interesting are Rs264 and Rs265, which represent two materials of the same variety but have different geographic origins and years of material collection, and although the two materials are clustered in the same population, they are genetically distant, suggesting that they actually represent are two different materials. We speculate that these results may be due to varietal mutations in response to environmental changes, or frequent introductions from various regions, leading to eventual confusion of the varietal names.

### Construction of SNP fingerprints

**Figure 5:**
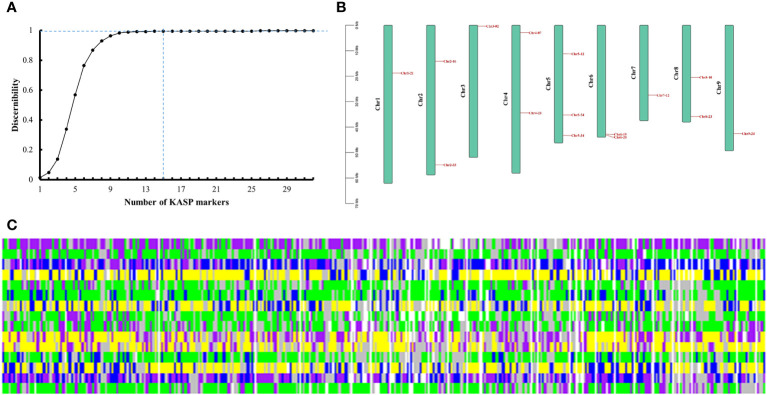
Fingerprint analysis of 356 radish varieties. **(A)** Identification efficiency of the combined SNP markers. **(B)** Distribution of 15 core markers on radish chromosomes. **(C)** Fingerprints of the 356 radish materials. Each row represents a genome and each column represents a sample. Pure genotypes are AA=yellow, CC=green, GG=blue, TT=purple; heterozygous genotypes are grey; no call genotypes are NA=white.

Among the 32 candidate markers, we calculated that the 356 radish varieties could be completely distinguished by 15 markers, which were evenly distributed in each chromosome of radish (**Figures 5A, B**). The average PIC value of these 15 markers was 0.34, which indicating high polymorphism, and the average sample no call rate was lower than 0.04. We believe that these 15 markers can be used as the core marker selection for the construction of fingerprints of the 356 radish varieties. We converted the genotypes identified by the 15 core markers into numerical coding, with pure genotypes represented by AA=1=yellow, CC=2=green, GG=3=blue, and TT=4=purple; heterozygous genotypes were converted to 5, with represented by the colour of grey; and the no call genotypes were represented by NA=6=white. Fingerprinting was carried out using R, with each row representing one SNP marker, and each column representing one sample (**Figure 5C**; **Supplementary Table 6**). Fingerprint analysis revealed that, Rs310 and Rs303, Rs312 and Rs317, Rs1618 and Rs25, Rs192 and Rs295, Rs94 and Rs176, and Rs18 and Rs24, differed only by a single SNP locus,and Rs184, Rs187, Rs304, Rs315, Rs318, Rs320 and Rs321 differed from each other only by their missing SNP loci, which we hypothesized might indicate that these radish materials represent the same variety; Rs113 and Rs323 performed similarly. The remaining varieties differed from each other by two or more SNP loci.

### Construction of core germplasm

Based on the 32 core SNP molecular marker sets obtained above, 71 representative core germplasm populations (**Supplementary Table 7**) of radish were constructed by combining Core Hunter software, which accounted for about 20% of the germplasm resources and the allele coverage rate reached 100%. For the mean genetic distance of the core germplasm population, we measured it using two indicators, namely the Rogers genetic distance (MR) and the Cavalli-Edwards genetic distance (CE), and the resulting genetic distances were 0.62 and 0.62, respectively. On this basis, we further calculated several genetic diversity indices of the core germplasm population, and obtained the shannon genetic diversity index (SH), expected heterozygosity (HE), and polymorphism information (PIC) of 4.11, 0.46, and 0.35, respectively (**Table 2**). Combine the above genetic diversity data, the core germplasm population showed certain diversity characteristics, which basically met the standard.

**Table 2:** Diversity analysis of radish core germplasm population.

Original germplasm populations	Core germplasm populations	MR	CE	SH	HE	NE	PIC	CV
356	71	0.62	0.62	4.12	0.46	1.86	0.35	100%

## Discussion

In recent years, the global production of radish has steadily increased, and the planting area has expanded, the annual production of radish in China ranks first in the world. To data, great efforts have been made worldwide in collecting radish germplasm resources, and more than 5000 radish varieties have been preserved globally, of which more than 2000 germplasm resources have been preserved in China (Kurina et al., 2021). With the expansion of market demand, the number of newly bred varieties is also increasing every year, leading to the phenomenon of homonymity, and infringement of breeders’ rights and interests. With the development of DNA molecular markers, different types, such as RAPD, AFLP, SSR, and InDel markers, have been used for the identification of radish germplasm resources, genetic diversity studies, and fingerprint construction (Pradhan et al., 2004; Kong et al., 2011; Bae et al., 2015; Li et al., 2023). However, these analytical markers have been gradually eliminated due to their poor stability, complex operation and low polymorphism. With the development of high-throughput sequencing, SNP markers due to their high number, wide distribution and good stability, are considered ideal for genetic studies of germplasm resources. With the application of KASP technology, SNP markers have been widely used in cruciferous vegetable crops such as cabbage (Li et al., 2019), kale (Li et al., 2020), cauliflower (Yang et al., 2022) and broccoli (Shen et al., 2021). However, no fingerprinting of radish based on the combination of SNP loci with KASP technology and genetic diversity analysis has been established thus far. Therefore, there is an urgent need to develop an accurate and efficient fingerprint database for the rapid  identification of radish varieties, seed purity testing, and population genetic analysis, which is of great significance for the conservation of germplasm resources and the promotion of industrial development.

In this study, we developed SNPs based on 520 published radish genomic data and prelaboratory resequencing genomic data from local cultivars around the world, demonstrating the reliability of these genomic data in their use for SNP development. Then, based on strict screening criteria, we ultimately obtained 308 high-quality SNPs evenly distributed on radish chromosomes. To validate the efficiency of high-quality SNP identification in radish, we selected 100 of the SNPs detected, of which 87 were successfully transformed into KASP markers (i.e., a transformation rate of 87%), and with screening based on KASP technology, we obtained a set of 32 candidate core markers for genetic analysis of 356 radish populations. To further obtain the core markers for the construction of radish fingerprints, we calculated the optimal combination by computer algorithms, and ultimately obtaining a dataset of 15 core markers that were used for the construction of the fingerprints of the 356 radish populations. We found that the core SNP markers developed in this experiment were highly polymorphic and stable. Compared with the 32 candidate core markers, using a subset of fewer core markers can provide a more rapid and convenient way to identify varieties, and at the same time reduce the corresponding cost loss, which is conducive to saving resources.

Radish germplasm resources are an important material basis for the selection and breeding of new radish varieties, genetic theory research and agricultural production (Kobayashi et al., 2020). Chinese radish germplasm resources are very rich, and the standardization and unification of their criteria are conducive to the collection and protection of radish germplasm resources, as well as the full exploitation of their potential economic value and research development (Kurina et al., 2021). Genetic analysis of population structure can improve the integration and utilization efficiency of germplasm resources, and many vegetable crops have been divided by population structure, Zhang et al. (2020) divided 261 Chinese cucumbers into four taxa: North China type, South China type, European type and Xishuangbanna type. North China type and Xishuangbanna type had lower genetic diversity, indicating that there is a greater risk of genetic erosion in these two subgroups; Shen et al. (2021) divided the population structure of 372 broccoli into two taxa, and the results showed that broccoli had a narrow genetic base, consisting of two subgroups with weak genetic structure; Li et al. (2020) divided the hybrid taxa of different kale self-inbreeding lines based on the data from KASP analysis, and analysed the genetic similarity between the self-inbreeding lines, which greatly improved the efficiency of kale breeding. In this study, we used 32 SNP markers to analyse the population structure and performed, principal component analysis and kinship studies of 356 radish varieties, and to divide them into two populations, which showed that there was a high risk of genetic erosion due to close gene exchange between the Pop-1 and Pop-2 populations. Therefore, the study of germplasm resources not only can improve breeding efficiency, but also provides better prospects for their conservation as well as their application in genetics.

Radish is an important cash crop among cruciferous vegetable crops, with cultivated radish grown in some countries such as China and the warm coasts of Eurasia, resulting in a narrow genetic background for radish; additionally, little research has been conducted in this area. In this study, we screened 15 core SNP markers to construct 356 radish fingerprints, and were able to completely differentiate all the materials, although some varieties were less genotypically distinct from each other. The results of this study provide an important theoretical basis for the conservation of radish germplasm resources, rational utilization of germplasm resources and selection and breeding of germplasm resources in China. On this basis, the current identification system will be further improved to enhance the accuracy and efficiency of identification. In the future, with the increasing number of new radish germplasm resources in China, it will be necessary to integrate more SNP markers and develop molecular markers that are closely linked to major agronomic traits.

With the extensive collection of germplasm resources, the germplasm resource bank has also been expanding, which greatly improves the management cost of the germplasm bank and the difficulty of screening specific germplasm. At the same time, breeders are unable to effectively evaluate and identify a large number of genetic materials, which will cause a large loss of germplasm and reduce the genetic diversity of germplasm over time (Lee et al., 2018). In this context, the concept of core germplasm has been proposed, which is to represent the richest genetic diversity, structure and geographical distribution of a species to the greatest extent with the least number of germplasm and the lowest genetic redundancy. It can help researchers quickly find germplasm with target traits, so as to efficiently evaluate, study and utilize germplasm resources (Li et al., 2023). In this study, we developed a core germplasm consisting of 71 radish materials accounting for 20% of the original germplasm population and 100% of the genetic diversity of the original germplasm. The construction of radish core germplasm can effectively protect the genetic diversity of radish germplasm and avoid the loss of resources, and can also be used as the basic material for breeding, providing important genetic resources for the breeding of new varieties, and contributing to the in-depth study of the genetic traits and ecological adaptability of radishes.

## Conclusion

In this study, we integrated published radish genomic data and genomic data completed by presequencing in the laboratory, developed SNP markers with reference to strict screening criteria, and combined them with KASP technology, to screen 32 candidate core SNPs for the genetic diversity analysis of 356 radish cultivars from local varieties from different parts of China as well as commercial varieties available in the market. We were able to classify the population into two groups and found that the two groups were genetically close to one another. At the same time, 15 core markers were screened and used to construct a fingerprint database of the 356 radish cultivars. The combination of markers was polymorphic and efficient, and could completely distinguish the 356 radish cultivars. In addition, we constructed a core germplasm population consisting of 71 radish materials, and the genetic diversity of this germplasm population covered 100% of the original germplasm population. Our results demonstrated the reliability and accuracy of DNA fingerprinting based on SNP markers in radish, providing technical support and promoting the development of molecular plant breeding for the identification of radish varieties, the delineation of relatedness, and the collection and conservation of germplasm resources.

## Data availability statement

The original contributions presented in the study are included in the article/**Supplementary Material**. Further inquiries can be directed to the corresponding author.

## Author contributions

XX: Data curation, Formal analysis, Investigation, Methodology, Project administration, Software, Validation, Visualization, Writing – original draft, Writing – review & editing. TH: Project administration, Resources, Writing – review & editing. YW: Funding acquisition, Resources, Writing – review & editing. YL: Funding acquisition, Resources, Writing – review & editing. WW: Writing – review & editing. HH: Writing – review & editing. QW: Writing – review & editing. YY: Writing – review & editing. DG: Writing – review & editing. CB: Funding acquisition, Project administration, Resources, Supervision, Writing – review & editing. JW: Data curation, Methodology, Project administration, Resources, Supervision, Writing – review & editing.

## Supplementary material

Supplementary Figure 1 Characteristics of the aerial parts of 46 parts of radish material.

Supplementary Figure 2 Characteristics of the underground part of 46 parts of radish material.

Supplementary Figure 3 Neighbour-joining(N-J) tree cluster analysis of 356 radish.

Supplementary Table 1 Introduction and source of 356 radish varieties.

Supplementary Table 2 46 radish varieties used for KASP marker screening.

Supplementary Table 3 32 candidate core SNP primers.

Supplementary Table 4 P-distance between 356 radish materials.

Supplementary Table 5 Genetic similarity coefficient matrix of 356 radish materials based on Nei1972.

Supplementary Table 6 Genotypic data for constructing fingerprints of 356 radish materials based on 15 core SNP markers.

Supplementary Table 7 71 core germplasm genotypes of radish.

## References

[B1] Aboul-Maaty N. A. F. Oraby H. A. S. (2019). Extraction of high-quality genomic DNA from different plant orders applying a modified CTAB-based method. Bull. Natl. Res. Centre 43 (1), 1–10. doi: 10.1186/s42269-019-0066-1

[B2] Bae K. M. Sim S. C. Hong J. H. Choi K. J. Kim D. H. Kwon Y. S. (2015). Development of genomic SSR markers and genetic diversity analysis in cultivated radish (*Raphanus sativus* L.). Horticulture Environment Biotechnol. 56, 216–224. doi: 10.1007/s13580-015-0089-y

[B3] Bradbury P. J. Zhang Z. Kroon D. E. Casstevens T. M. Ramdoss Y. Buckler E. S. (2007). TASSEL: software for association mapping of complex traits in diverse samples. Bioinformatics 23 (19), 2633–2635. doi: 10.1093/bioinformatics/btm308 17586829

[B4] Button P. (2007). The international union for the protection of new varieties of plants (UPOV) recommendations on variety denominations. V Int. Symposium Taxonomy Cultivated Plants 799, 191–200. doi: 10.17660/actahortic.2008.799.27

[B5] Chen H. Xie W. He H. Yu H. Chen W. Li J.. (2014). A high-density SNP genotyping array for rice biology and molecular breeding. Mol. Plant 7, 541–553. doi: 10.1093/mp/sst135 24121292

[B6] Chen S. Zhou Y. Chen Y. Gu J. (2018). Fastp: an ultra-fast all-in-one FASTQ preprocessor. Bioinformatics 34 (17), i884–i890. doi: 10.1093/bioinformatics/bty560 30423086 PMC6129281

[B7] Chen Z. Tang D. Ni J. Li P. Wang L. Zhou J.. (2021). Development of genic KASP SNP markers from RNA-Seq data for map-based cloning and marker-assisted selection in maize. BMC Plant Biol. 21, 1–11. doi: 10.1186/s12870-021-02932-8 33771110 PMC8004444

[B8] Dreisigacker S. Sharma R. K. Huttner E. Karimov A. Obaidi M. Q. Singh P. K.. (2019). Tracking the adoption of bread wheat varieties in Afghanistan using DNA fingerprinting. BMC Genomics 20 (1), 1–13. doi: 10.1186/s12864-019-6015-4 31426740 PMC6699131

[B9] Evanno G. Regnaut S. Goudet J. (2005). Detecting the number of clusters of individuals using the software STRUCTURE: a simulation study. Mol. Ecol. 14, 2611–2620. doi: 10.1111/j.1365-294X.2005.02553.x 15969739

[B10] Falush D. Stephens M. Pritchard J. K. (2003). Inference of population structure using multilocus genotype data: linked loci and correlatedallele frequencies. Genetics 164, 1567–1587. doi: 10.3410/f.1015548.19 12930761 PMC1462648

[B11] Grewal S. Hubbart-Edwards S. Yang C. Devi U. Baker L. Heath J.. (2020a). Rapid identification of homozygosity and site of wild relative introgressions in wheat through chromosome-specific KASP genotying assays. Plant Biotechnol. J. 18 (3), 743–755. doi: 10.1111/pbi.13241 31465620 PMC7004896

[B12] Grewal S. Othmeni M. Walker J. Hubbart-Edwards S. Yang C. Y. Scholefield D.. (2020b). Development of wheat-Aegilops caudata introgression lines and their characterization using genome-specific KASP markers. Front. Plant Sci. 11. doi: 10.3389/fpls.2020.00606 PMC7240103 32477394

[B13] Hong H. Lee J. Chae W. (2023). An economic method to identify cultivars and elite lines in radish (*Raphanus sativus* L.) for small seed companies and independent breeders. Horticulturae 9 (2), 140. doi: 10.3390/horticulturae9020140

[B14] Huang J. Yang L. Yang L. Wu X. Cui X. Zhang L.. (2023). Stigma receptors control intraspecies and interspecies barriers in *Brassicaceae* . Nat. 614 (7947), 303–308. doi: 10.1038/s41586-022-05640-x PMC9908550 36697825

[B15] Iqbal M. Z. Jamil S. Shahzad R. Bilal K. Qaisar R. Nisar A.. (2021). DNA fingerprinting of crops and its applications in the field of plant breeding. J. Agric. Res. 59 (1), 13–28. doi: 10.1007/978-3-0348-7312-3_21

[B16] Jamali S. H. Cockram J. Hickey L. T. (2019). Insights into deployment of DNA markers in plant variety protection and registration. Theor. Appl. Genet. 132, 1911–1929. doi: 10.1007/s00122-019-03348-7 31049631

[B17] Jamil S. Shahzad R. Iqbal M. Z. Yasmeen E. Rahman S. U. (2021). DNA fingerprinting and genetic diversity assessment of GM cotton genotypes for protection of plant breeders rights. Int. J. Agric. Biol. 25 (4), 768–776. doi: 10.17957/IJAB/15.1728

[B18] Kang E. S. Ha S. M. Ko H. C. Yu H. J. Chae W. B. (2016). Reproductive traits and molecular evidence related to the global distribution of cultivated radish (*Raphanus sativus* L.). Plant Systematics Evol. 302, 1367–1380. doi: 10.1007/s00606-016-1336-0

[B19] Kobayashi H. Shirasawa K. Fukino N. Hirakawa H. Akanuma T. Kitashiba H. (2020). ). Identification of genome-wide single-nucleotide polymorphisms among geographically diverse radish accessions. DNA Res. 27 (1), dsaa001. doi: 10.1093/dnares/dsaa001 32065621 PMC7315352

[B20] Kong Q. Li X. Xiang C. Wang H. Song J. Zhi H. (2011). Genetic diversity of radish (Raphanus sativus L.) germplasm resources revealed by AFLP and RAPD markers. Plant Mol. Biol. Rep. 29, 217–223. doi: 10.1007/s11105-010-0228-7

[B21] Kumar S. Stecher G. Li M. Knyaz C. Tamura K. (2018). MEGA X: molecular evolutionary genetics analysis across computing platforms. Mol. Biol. Evol. 35 (6), 1547. doi: 10.1093/molbev/msy096 29722887 PMC5967553

[B22] Kurina A. B. Kornyukhin D. L. Solovyeva A. E. Artemyeva A. M. (2021). Genetic diversity of phenotypic and biochemical traits in VIR radish (*Raphanus sativus* L.) germplasm collection. Plants 10 (9), 1799. doi: 10.3390/plants10091799 34579332 PMC8468841

[B23] Lee Y. J. Mun J. H. Jeong Y. M. Joo S. H. Yu H. J. (2018). Assembly of a radish core collection for evaluation and preservation of genetic diversity. Horticulture Environment Biotechnol. 59, 711–721. doi: 10.1007/s13580-018-0079-y

[B27] Li X. Cui L. Zhang L. Huang Y. Zhang S. Chen W.. (2023). Genetic diversity analysis and core germplasm collection construction of radish cultivars based on structure variation markers. Int. J. Mol. Sci. 24 (3), 2554. doi: 10.3390/ijms24032554 36768875 PMC9916615

[B24] Li H. Durbin R. (2009). Fast and accurate short read alignment with Burrows–Wheeler transform. bioinformatics 25 (14), 1754–1760. doi: 10.1093/bioinformatics/btp324 19451168 PMC2705234

[B25] Li H. Handsaker B. Wysoker A. Fennell T. Ruan J. Homer N.. (2009). 1000 Genome Project Data Processing Subgroup. 2009. The sequence alignment/map format and samtools. Bioinformatics 25 (16), 2078–2079. doi: 10.1093/bioinformatics/btp352 19505943 PMC2723002

[B26] Li P. Su T. Yu S. Wang H. Wang W. Yu Y.. (2019). Identification and development of a core set of informative genic SNP markers for assaying genetic diversity in Chinese cabbage. Horticulture Environment Biotechnol. 60, 411–425. doi: 10.1007/s13580-019-00138-4

[B28] Li X. Wang J. Qiu Y. Wang H. Wang P. Zhang X.. (2021). SSR-sequencing reveals the inter-and intraspecific genetic variation and phylogenetic relationships among an extensive collection of Radish (*Raphanus*) germplasm resources. Biology 10 (12), 1250. doi: 10.3390/biology10121250 34943165 PMC8698774

[B29] Li Z. Yu H. Li X. Zhang B. Ren W. Liu X.. (2020). Kompetitive allele-specific PCR (KASP) genotying and heterotic group classification of 244 inbred lines in cabbage (*Brassica oleracea* L. var. *capitata*). Euphytica 216, 1–11. doi: 10.1007/s10681-020-02640-8

[B30] Liu K. Muse S. V. (2005). PowerMarker: an integrated analysis environment for genetic marker analysis. Bioinformatics 21 (9), 2128–2129. doi: 10.1093/bioinformatics/bti282 15705655

[B31] McKenna A. Hanna M. Banks E. Sivachenko A. Cibulskis K. Kernytsky A.. (2010). The Genome Analysis Toolkit: a MapReduce framework for analyzing next-generation DNA sequencing data. Genome Res. 20 (9), 1297–1303. doi: 10.1101/gr.107524.110 20644199 PMC2928508

[B32] Nadeem M. A. Nawaz M. A. Shahid M. Q. Doğan Y. Comertpay G. Yıldız M.. (2018). DNA molecular markers in plant breeding: current status and recent advancements in genomic selection and genome editing. Biotechnol. Biotechnol. Equip. 32 (2), 261–285. doi: 10.1080/13102818.2017.1400401

[B33] Noman O. M. Nasr F. A. Alqahtani A. S. Al-Zharani M. Cordero M. A. W. Alotaibi A. A.. (2021). Comparative study of antioxidant and anticancer activities and HPTLC quantification of rutin in white radish (*Raphanus sativus* L.) leaves and root extracts grown in Saudi Arabia. Open Chem. 19 (1), 408–416. doi: 10.1515/chem-2021-0042

[B34] Nybom H. Weising K. Rotter B. (2014). DNA fingerprinting in botany: past, present, future. Invest. Genet. 5 (1), 1–35. doi: 10.1186/2041-2223-5-1 PMC3880010 24386986

[B35] Ongom P. O. Fatokun C. Togola A. Salvo S. Oyebode O. G. Ahmad M. S.. (2021). Molecular fingerprinting and hybridity authentication in cowpea using single nucleotide polymorphism based kompetitive allele-specific PCR assay. Front. Plant Sci. 12. doi: 10.3389/fpls.2021.734117 PMC8524091 34675950

[B36] Pearse I. S. Bastow J. L. Tsang A. (2014). Radish introduction affects soil biota and has a positive impact on the growth of a native plant. Oecologia 174, 471–478. doi: 10.1007/s00442-013-2779-4 24072439

[B37] Petr D. Auton A. Goncalo A. Cornelis A. Banks E. Mark A. (2011). 1000 genomes project analysis group. The variant call format and VCF tools. Bioinformatics 27 (15), 2156–2158. doi: 10.1093/bioinformatics/btr330 21653522 PMC3137218

[B38] Pradhan A. Yan G. Plummer J. A. (2004). Development of DNA fingerprinting keys for the identification of radish cultivars. Aust. J. Exp. Agric. 44 (1), 95–102. doi: 10.1071/EA03031

[B39] Satturu V. Rani D. Gattu S. Md J. Mulinti S. Nagireddy R. K.. (2018). DNA fingerprinting for identification of rice varieties and seed genetic purity assessment. Agric. Res. 7 (4), 379–390. doi: 10.1007/s40003-018-0324-8

[B40] Selvakumar R. (2022). An update on radish breeding strategies: an overview. Case Studies of Breeding Strategies in Major Plant Species 14, 1–26. doi: 10.5772/intechopen.108725

[B41] Semagn K. Babu R. Hearne S. Olsen M. (2014). Single nucleotide polymorphism genotyping using Kompetitive Allele Specific PCR (KASP): overview of the technology and its application in crop improvement. Mol. Breed. 33, 1–14. doi: 10.1007/s11032-013-9917-x

[B43] Shen Y. Wang J. Shaw R. K. Yu H. Sheng X. Zhao Z.. (2021). Development of GBTS and KASP panels for genetic diversity, population structure, and fingerprinting of a large collection of broccoli (Brassica oleracea L. var. italica) in China. Front. Plant Sci. 12. doi: 10.3389/fpls.2021.655254 PMC8213352 34149754

[B44] Sucher N. J. Hennell J. R. Carles M. C. (2012). DNA fingerprinting, DNA barcoding, and next generation sequencing technology in plants. Plant DNA fingerprinting barcoding: Methods Protoc. 862, 13–22. doi: 10.1007/978-1-61779-609-8_2 22419485

[B45] Thachuk C. Crossa J. Franco J. Dreisigacker S. Warburton M. Davenport G. F. (2009). Core Hunter: an algorithm for sampling genetic resources based on multiple genetic measures. BMC Bioinf. 10, 243. doi: 10.1186/1471-2105-10-243 PMC2734557 19660135

[B46] Tian H. Yang Y. Wang R. Fan Y. Yi H. Jiang B.. (2021). Screening of 200 core SNPs and the construction of a systematic SNP-DNA standard fingerprint database with more than 20,000 maize varieties. Agriculture 11 (7), 597. doi: 10.3390/agriculture11070597

[B47] Tian H. Wang F. Zhao J. Yi H. Wang L. Wang R.. (2015). Development of maizeSNP3072, a high-throughput compatible SNP array, for DNA fingerprinting identification of Chinese maize varieties. Mol. Breed. 35, 1–11. doi: 10.1007/s11032-015-0335-0 PMC4449932 26052247

[B48] Wang F. Fan X. Zhang Y. Lei S. Liu C. Jiang J. (2022). Establishment and application of an SNP molecular identification system for grape cultivars. J. Integr. Agric. 21 (4), 1044–1057. doi: 10.1016/S2095-3119(21)63654-7

[B49] Wang H. Li X. Song J. (2018). Vegetable genetic resources in China[J]. Hortic. Plant J. 4 (2), 83–88. doi: 10.1016/j.hpj.2018.03.003

[B51] Wang Y. Lv H. Xiang X. Yang A. Feng Q. Dai P.. (2021). Construction of a SNP fingerprinting database and population genetic analysis of cigar tobacco germplasm resources in China. Front. Plant Sci. 12. doi: 10.3389/fpls.2021.618133 PMC7943628 33719288

[B50] Wang L. Xun H. Aktar S. Zhang R. Wu L. Ni D.. (2022). Development of SNP markers for original analysis and germplasm identification in camellia sinensis. Plants 12 (1), 162. doi: 10.3390/plants12010162 36616292 PMC9824298

[B52] Wei Q. Wang J. Wang W. Hu H. Yan Y. Bao C.. (2022). Identification of QTLs controlling radish root shape using multiple populations. Horticulturae 8, 931. doi: 10.3390/horticulturae8100931

[B53] Yang G. Chen S. Chen L. Sun K. Huang C. Zhou D.. (2019). Development of a core SNP arrays based on the KASP method for molecular breeding of rice. Rice 12, 1–18. doi: 10.1186/s12284-019-0272-3 30963280 PMC6453994

[B54] Yang Y. Lyu M. Liu J. Wu J. Wang Q. Xie T.. (2022). Construction of an SNP fingerprinting database and population genetic analysis of 329 cauliflower cultivars. BMC Plant Biol. 22 (1), 1–11. doi: 10.1186/s12870-022-03920-2 36357859 PMC9647966

[B56] Zhang X. Liu T. Wang J. Wang P. Qiu Y. Zhao W.. (2021). Pan-genome of Raphanus highlights genetic variation and introgression among domesticated, wild, and weedy radishes. Mol. Plant 14 (12), 2032–2055. doi: 10.1016/j.molp.2021.08.005 34384905

[B55] Zhang J. Yang J. Zhang L. Luo J. Zhao H. Zhang J.. (2020). A new SNP genotying technology Target SNP-seq and its application in genetic analysis of cucumber varieties. Sci. Rep. 10 (1), 5623. doi: 10.1038/s41598-020-62518-6 32221398 PMC7101363

