# Supplementary figures and images for: Construction of SNP fingerprints and genetic diversity analysis of radish (Raphanus sativus L.)
Source: Front Plant Sci. 2024 Feb 2;15:1329890. doi: 10.3389/fpls.2024.1329890 (PMC10869463; doi:10.3389/fpls.2024.1329890)

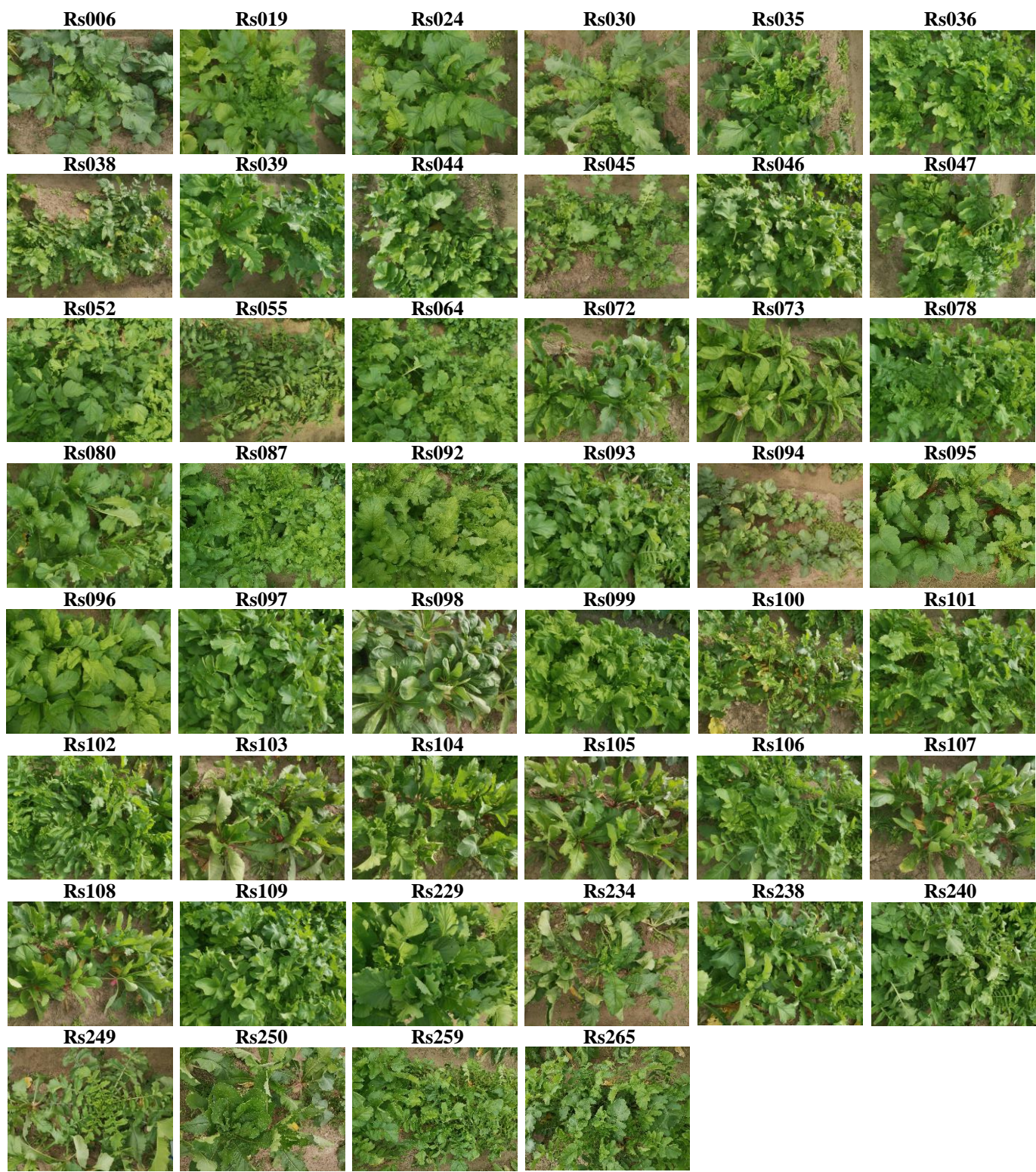

Supplement: Supplementary Figure 1 — Characteristics of the aerial parts of 46 parts of radish material. [file DataSheet_1.pdf]

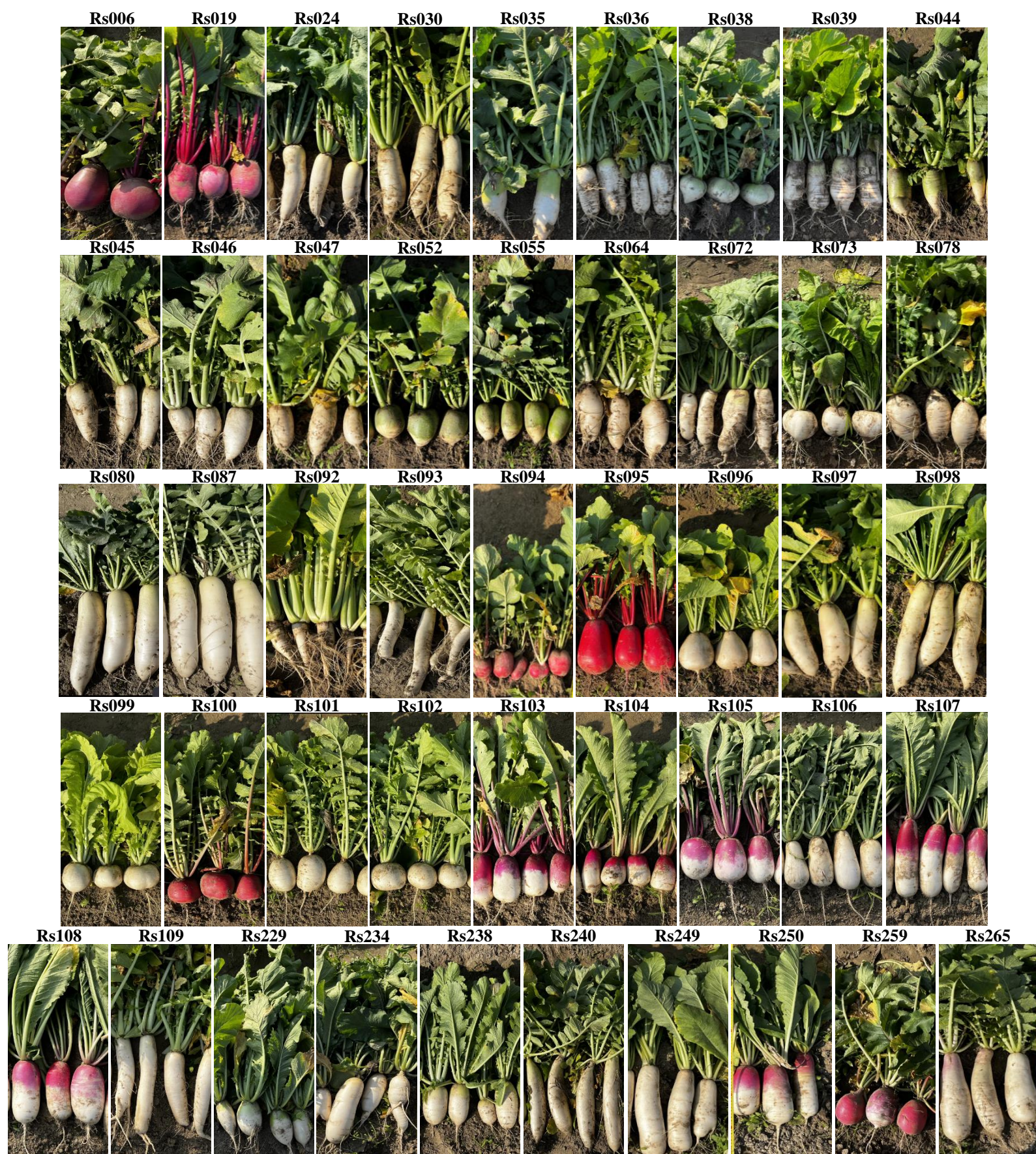

Supplement: Supplementary Figure 2 — Characteristics of the underground part of 46 parts of radish material. [file DataSheet_2.pdf]

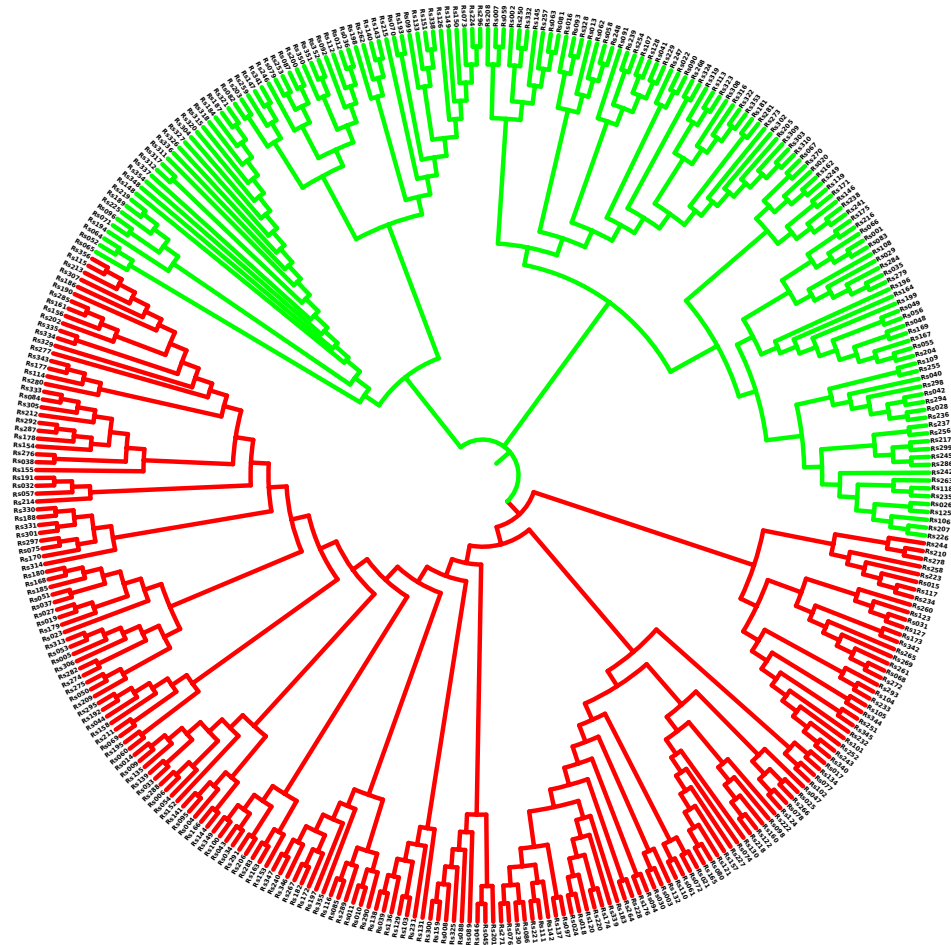

Supplement: Supplementary Figure 3 — Neighbour-joining(N-J) tree cluster analysis of 356 radish. [file DataSheet_3.pdf]
